# Perceptual grouping and the bounce–stream illusion

**DOI:** 10.1177/20416695251341689

**Published:** 2025-06-17

**Authors:** Nihan Alp, Stuart Anstis

**Affiliations:** 1 Department of Psychology, 52991 Sabanci University, Istanbul, Turkey; 2 Department of Psychology, University of California, San Diego, La Jolla, CA, USA

**Keywords:** bounce, stream, bistable, motion, perception

## Abstract

Two spots moving simultaneously along the same path in opposite directions can appear either to bounce or stream. Bouncing is promoted by spots that turn back and retrace their path. Streaming is promoted by same-size spots, moving along a straight path and viewed peripherally. Both percepts are driven by Gestalt grouping.

## How to cite this article

Alp, N., & Anstis, S. (2025). Perceptual grouping and the bounce–stream illusion, *i–Perception, 16(0)*, 1–6. https://doi.org/10.1177/20416695251341689

Bounce–stream is an ambiguous motion stimulus in which two objects move along the same path in opposite directions. When they meet, they either ***stream*** past each other unscathed, or they collide and ***bounce*** back to their starting points. Many variables affect the bounce–stream, including sounds around the moment of overlap (Sekuler et al., 1997), interruption of the objects’ motion, tactile stimulation, expectation/attention (Kawachi et al., 2011; Vroomen & Keetels 2020), and the language used in the instructions (Cui & Ariga, 2020). But here we confine ourselves to the influence of **perceptual grouping** on bounce–stream.

If the two moving objects are identical, their default is to stream, making the motion continue in the same direction (common fate). But nonidentical objects are paired off or perceptually grouped (either bounce or stream), based on their *similarity* of color, contrast, shape, or size.

**Figure 1.**
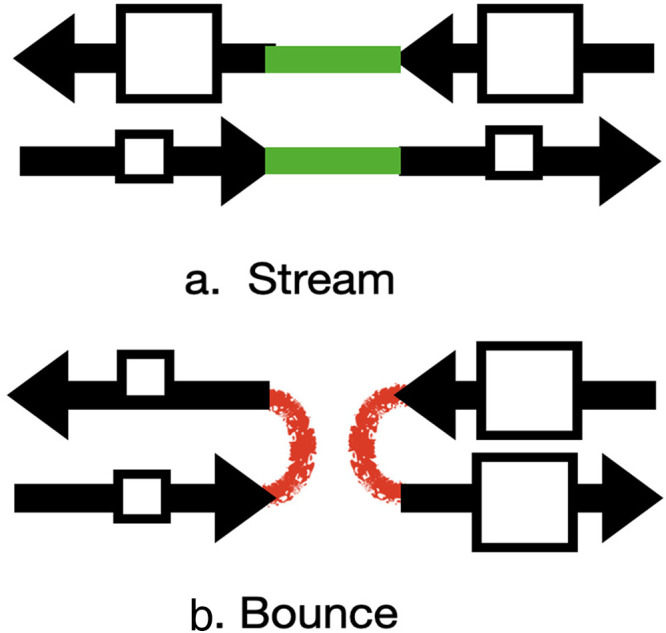
(a) Spots Stream and (b) Spots Bounce. Black Arrows Show the Stimuli, and Red and Green Lines Show the Perceptual Groupings.

Thus, Figure 1a shows two squares of different sizes that *stream* past each other along straight paths. In Figure 1b, the squares switch sizes (or other form qualities) at the midline. They *bounce*, so that all squares to the left of the collision point are small and all squares to the right of the collision point are large.

Two textured squares of average size 2° moved in opposite directions at a speed of 0.75°/s along a horizontal path 13° in length. The two squares were of different sizes on different trials, and these size changes *always promoted bouncing*, as shown in Figure 1b (squares to the left of the midpoint were always small and to the right were larger). On each trial, the ratio in the linear sizes of the two squares was set randomly to a value chosen from 1, 1.1, 1.22, 1.38, 1.5, 1.67, 1.86, and 2.08. Thus, for a size ratio = 1, both squares were 2° in size while for a size ratio = 2, the squares were 1.3° and 2.6° in size.

**Figure 2.**
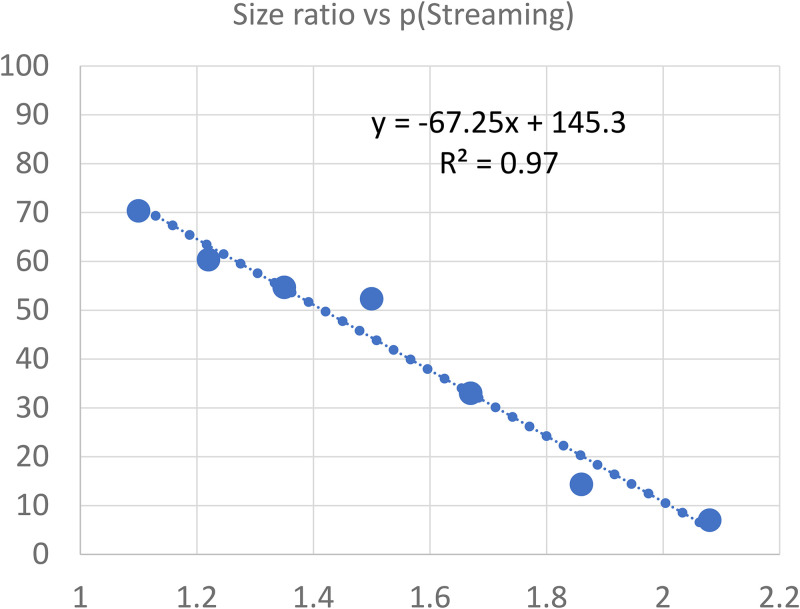
Streaming Decreased Linearly as the Squares Differed More in Size (*N* = 3).

In Experiment 1, the observers (*N* = 3) fixated a point 4° below the midpoint and pressed designated keys to indicate a perceived bounce or stream. All key presses were recorded for later analysis. Experiment 1 consisted of 147 trials (7 size ratios × 3 observers × 7 trials). Results, as plotted in Figure 2, show that when one square was 50% larger than the other (size ratio = 1.5) there was a 50-50 chance of its bouncing or streaming. Squares that were more similar in size (ratio < 1.5) usually streamed and squares that differed more in size (ratio > 1.5) usually bounced. If one square was twice the size of the other, it always bounced.

*Eccentricity.* We noticed that squares and rectangles that bounced in foveal vision often streamed when viewed in the periphery. Therefore, we repeated these observations at different eccentricities in Experiment 2, with size change again set to encourage bouncing. The two squares were randomly assigned to different sizes as before. The observers slowly moved the mouse cursor downwards, starting from the collision point, and fixating on it steadily (increasing the eccentricity), until the squares appeared to change from bouncing to streaming. In Experiment 2, the observers (*N* = 3) clicked the mouse to record this eccentricity and the next trial began. This was repeated for tall [and wide] rectangles that were the same height [width] as the squares but with a fixed width [length] of 0.5°. Experiment 2 consisted of 216 trials (8 size ratios × 3 shapes × 3 observers × 3 trials) (Movie 1).

**Movie 1.** Fixate These Unequal Squares and they Bounce. Now View them Peripherally and they Stream.

**Figure 3.**
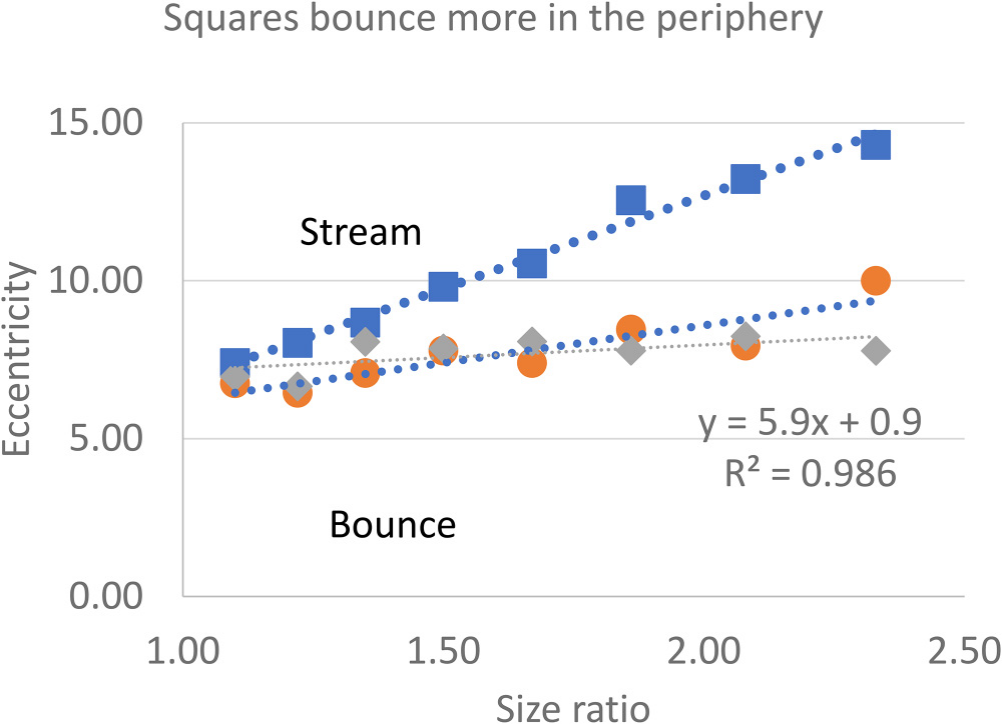
Tall (○) or Wide (◊) Rectangles Switched from Bounce to Stream at Eccentricities of 7°−10° Regardless of their Dimensions. But for Squares (□), the More Unequal the Two Squares (High Size Ratio), the More Bouncing Persists Even into Greater Eccentricities.

*Results*, as plotted in Figure 3, show that tall rectangles (○) and wide rectangles (◊) switched from bounce to stream at a constant eccentricity of about 7° regardless of their dimensions. But for squares (□) the greater the size difference the more the squares bounced, requiring greater eccentricity to switch from bouncing to streaming. Thus, squares were likely to bounce depending more on their eccentricity than on their dimensions, whereas for rectangles it depended both on their eccentricity and on their size ratios. The reason is that doubling the size ratio to encourage bouncing doubled the area of the rectangles but quadrupled the area of the squares.

*The paradox of bent paths*. So far, the two squares always moved in opposite directions along a perfectly straight line. In Experiment 3 (*N* = 4), now we used discs of diameter 1° whose paths met at an angle between 10° and 90°. Experiment 3 consisted of 160 trials (8 angles × 4 observers × 5 trials) (Movie 2).

**Movie 2.** Spots on a 90° Bent Path *Bounce*, But Adding a Size Change Makes their *Stream*.

**Figure 4.**
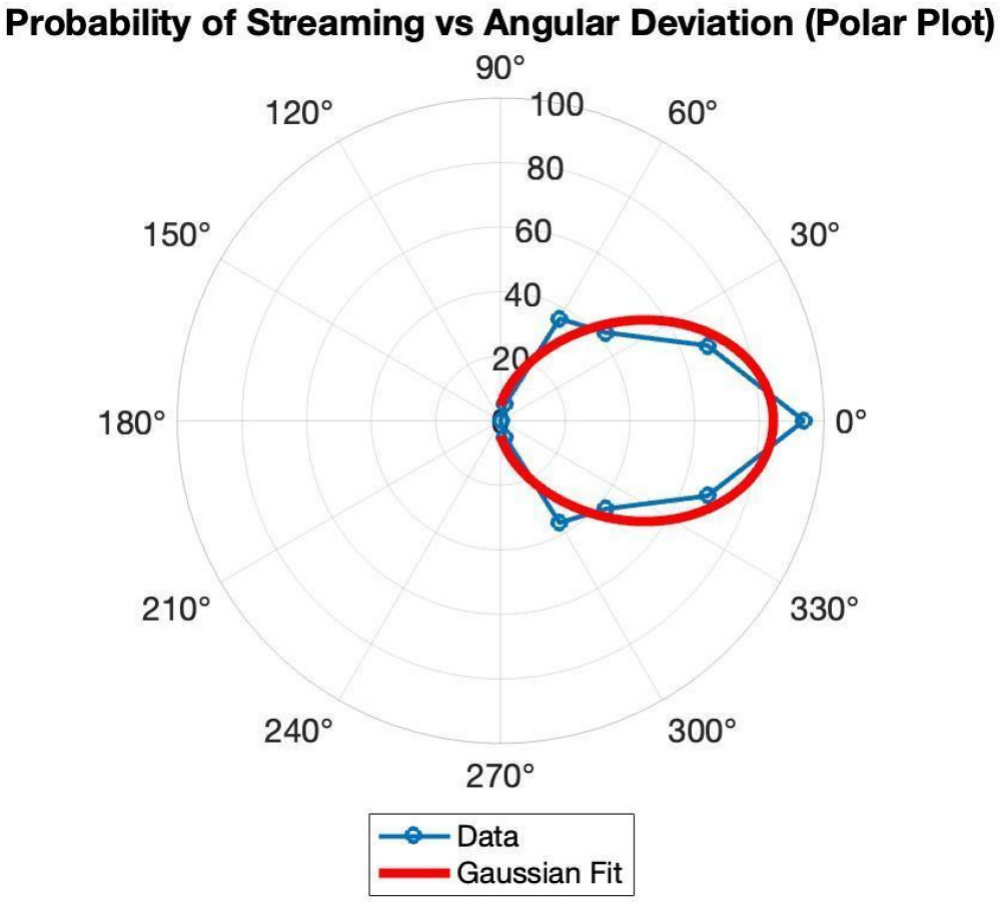
The Bend in the Path (0°–360°) is Shown Around the Circular Axis, Where 0° = Motion Along a Straight Path. The Length of the Radii Represents the Probability of Perceiving Streaming. Results Show that the Straighter the Motion Path the More the Streaming.

Results, in Figure 4, show that straight-line motion was nearly always perceived as streaming, but the probability of streaming fell off steadily for greater deviations, falling to almost zero for 90°. Paradoxically, a 90° bend would have made streaming spots change their direction by 90°. But this was never seen. Instead, the spots always bounced, changing their direction by no < 180°

Data were fitted as follows:

$$P(\theta )\;=a*\exp\left(-\frac{{\left(\theta -b\right)}^{2}}{2c^{2}}\right)$$

Here, *P*(*θ*) is the probability of streaming, *θ* is the bend angle, *a* is the peak probability, *b* is the mean of the Gaussian distribution, and *c* is the standard deviation.

**Figure 5.**
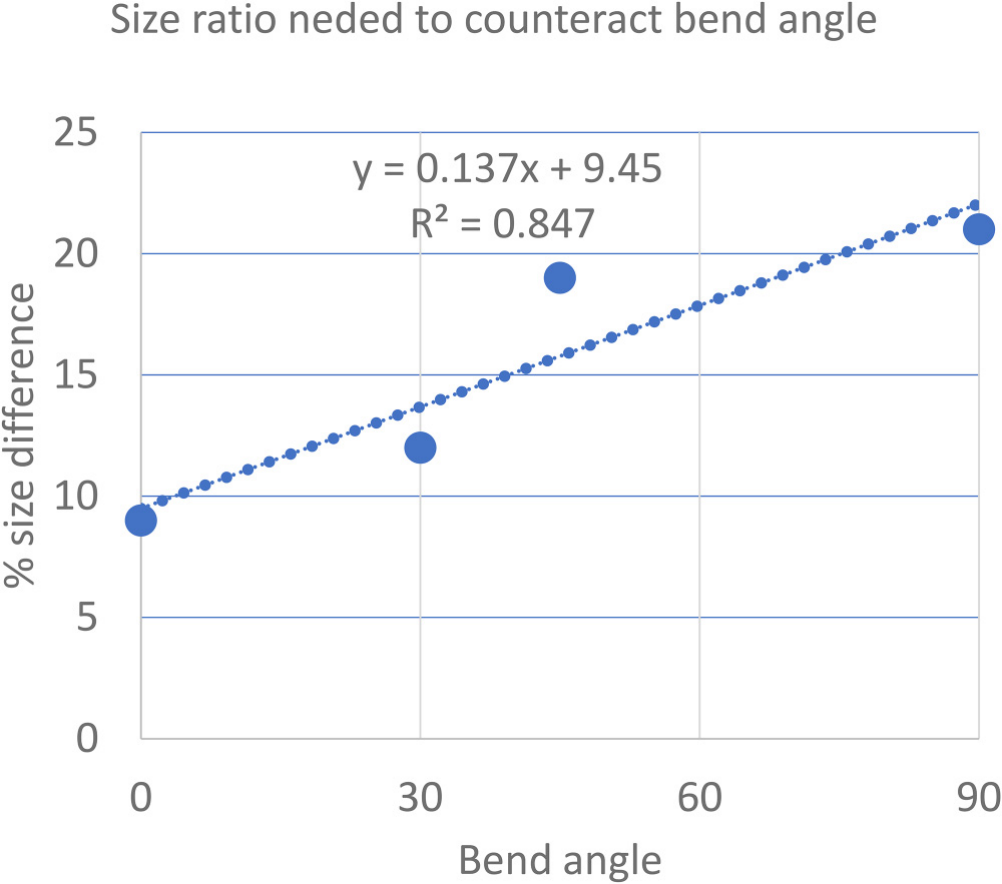
A Bend Angle of 90° Could be Changed from Bouncing to Streaming by a Size Difference of ∼22% (*N* = 4).

In Experiment 4, bent-path bouncing can switch to streaming if the two discs differ sufficiently in size (Figure 5 and Movie 2) (*N* = 4). Experiment 4 consisted of 160 trials (8 bend angles × 4 observers × 5 trials).

Lastly, Movie 3 gives a data-free demonstration.

**Movie 3.** Luminance Polarity Favors Bouncing or Streaming.

## Conclusion

Our results show that Gestalt principles of grouping (similarity, common fate, etc.) that were developed for large arrays of static targets, also apply to the very different perceptual task of rapidly matching up pairs of moving targets on the fly.
